# Effect of puerarin on action potential and sodium channel activation in human hypertrophic cardiomyocytes

**DOI:** 10.1042/BSR20193369

**Published:** 2020-02-14

**Authors:** Yu-hui Lin, Xiao-Bin Ni, Jian-wu Zhang, Cai-wen Ou, Xiao-qing He, Wen-jun Dai, Xi-ming Chen, Min-sheng Chen

**Affiliations:** 1 Department of Cardiovascular Medicine, The Third Affiliated Hospital of Guangzhou Medical University, Guangzhou, China; 2 Department of Cardiovascular Medicine, The First Affiliated Hospital of Shantou University Medical College, Shantou, China; 3 Department of Cardiovascular Medicine, Nanfang Hospital, Southern Medical University, Guangzhou, China; 4 Department of Cardiovascular Medicine, Zhujiang Hospital, Southern Medical University, Guangzhou, China

**Keywords:** Action potential, Cardiomyocytes, Induced pluripotent stem cells, Ion channel, Puerarin

## Abstract

Objective**:** To study the effect of puerarin on electrophysiology using a hypertrophic cardiomyocyte (HC) model.

Materials and methods: Human urine epithelial cells were used to generate the HC model (hiPSC-CM). Cardiomyocyte hypertrophy was induced by applying 10 nM endothelin-1 (ET-1). Effects of puerarin pre-treatment (PPr) and post-treatment (PPo) on action potential, sodium current (*I*_Na_) activation and inactivation, and recovery following *I*_Na_ inactivation were tested using patch clamp electrophysiology.

Results: Depolarization to repolarization 50% time (APD50) and repolarization 30% time (APD30) were significantly prolonged in the PPo and PPr groups compared with the controls. However, there were no significant differences in the action potential depolarization amplitude (APA) or the maximum depolarization velocity (*V*_max_) in phase 0. The PPr group had a slightly shortened APD90, and an extended APD50 and APD30, but did not exhibit any significant changes in stage A of APA and *V*_max_. The PPo group did not exhibit any significant changes in I_Na_, while 12 h of PPr improved *I*_Na_. However, puerarin did not significantly affect the activation, inactivation, or recovery of the sodium channel.

Conclusions: Cardiomyocyte hypertrophy significantly decreased the *V*_max_ of the action potential and the peak density of *I*_Na_. PPr inhibited the decrease in *V*_max_ and increased the peak density of *I*_Na_. Thus, puerarin could be used to stabilize the electrophysiological properties of hypertrophic cardiomyocytes and reduce arrhythmias.

## Introduction

Cardiac hypertrophy refers to abnormal cardiac enlargement or thickness of the cardiac muscle. Cardiac hypertrophy is a compensatory mechanism that can be caused by physiological (exercise) or pathophysiological (hypertension or valvular disease) changes, resulting in altered cardiomyocyte size and extracellular matrix production. Prolonged pathophysiological hypertrophy can damage cardiomyocyte elasticity, potentially resulting in heart failure. Cardiomyocyte decompensation, as well as decreased systolic and diastolic function, can also alter cardiomyocyte electrophysiological activity, thereby inducing the onset of arrhythmia [1].

Endothelin-1 (ET-1) is a 21 amino acid polypeptide that is a potent vasoconstrictor secreted by vascular endothelial cells and cardiomyocytes [2,3]. Recent studies have shown that ET-1 levels in the myocardium are higher compared with levels in the circulation. ET-1 also varies compared with other myocardium hormones, such as angiotensin II, isoproterenol, growth hormone, and insulin-like growth factor-1. Thus, ET-1 is more likely to be a local regulator than a systemic hormone [4]. ET-1 induces hypertrophic responses by activating fetal gene expression and promoting protein synthesis and accumulation to increase cell surface area and cell size. The signaling pathway induced by ET-1 in cardiac hypertrophy involves activation of phospholipase C (PLC), protein kinase C (PKC), phospholipase A2 (PLA2), Jun N-terminal kinase (JNK), and mitogen-activated protein kinase (MAPK) [4,5].

Puerarin [7-hydroxy-3-(4-hydroxyphenyl)-1-benzopyran-4-one 8-(β-D-gluco-pyranoside)] is an isoflavone extracted from the root of the leguminous creeper Pueraria (Radix puerariae) and can also be found in several plants and herbs, especially in the kudzu plant [6]. Puerarin has multiple pharmacological properties and has been used to treat cardiovascular disorders, such as myocardial infarction and hypertensive angina pectoris [7]. Puerarin confers cardio protection by inhibiting inward rectifier potassium current and inhibiting the L-type calcium channel [8–10].

Adult somatic cells have been successfully induced into pluripotent stem cells (hiPSCs), which are derived from autologous cells [11–13], and can be differentiated into many cell types, including cardiomyocytes (hiPSC-CMs) [14]. hiPSC-CMs display various pharmacological and electrophysiological properties of human cardiomyocytes, including the ability to generate action potentials and respond to anti-arrhythmia drugs [15,16]. Previous studies have primarily used human embryonic kidney cells, Xenopus oocytes, and Chinese hamster ovary (CHO) cells for ion channel studies. However, these heterologous expression systems often lack the macromolecular complexes required to form ion channels in human cardiomyocytes. The integrity of the ion channel structure is essential for normal electrophysiological activity. The electrophysiological properties of the myocardium in transgenic animals are also different from those in human myocardium. However, hiPSC-CMs can genocopy and phenocopy properties of human hereditary heart disease. Therefore, given the difficulty in obtaining human ventricular myocytes, hiPSC-CMs are a good model to study human myocardial electrophysiological activity [17].

In view of the significant increase in the incidence of arrhythmia after cardiac hypertrophy, there are limited therapeutic options to prevent or treat arrhythmias. Therefore, we investigated the effects of puerarin on action potential geneation and sodium channel activity in ET-1-induced hypertrophic cardiomyocytes.

## Material and methods

### Generation of hiPSCs

hiPSCs were prepared from primary human epithelial cells obtained from the urine of the healthy donor using the Sendai virus (Invitrogen, Groningen, The Netherlands), which carries reprogramming factors [sex determining region Y-box 2 (SOX2), octamer-binding transcription factor 4 (OCT4), Kruppel-like factor 4 (KLF4) and c-MYC], as previously described [18,19]. To verify if the isolated hiPSCs were pluripotent, we stained the cells with the immunofluorescence marker Alkaline Phosphatase and measured teratoma formation.

### hiPSC-CM differentiation

The hiPSCs were cultured in an incubator with 5% CO_2_ at 37°C in CardioEasy Human Cardiomyocyte Differentiation Complete Medium I (CEHCDCM) (CELLAPY, Beijing, China) for 48 h, followed by CHCD complete medium III (CELLAPY, Beijing, China) for 24 h, and then CEHCDCM II (CELLAPY, Beijing, China) for 48 h. Differentiation started once the confluence rate of hiPSC-CMs reached 90%. The non-cardiomyocytes gradually died in the CardioEasy Human Myocardial Purified Complete Medium (CEHMPCM) (CELLAPY, Beijing, China), purifying the differentiated cultured cardiomyocytes (hiPSC-CMs). Following hiPSC differentiation, the culture medium was replaced for further testing.

### Identification of myocardial specific markers

α-Actinin (Cell Signaling, Danvers, MA, U.S.A.) and troponin T2 (TNNT2) (Abeam, Cambridge, U.K.) expression in hiPSC-CMs were detected using immunofluorescence. Expression of OCT4, NANOG, NK2 homeobox 5 (NKX2-5), myosin light chain 2 (MYL2), α-cardiac myosin heavy chain (MYH6), and MYH7 in the differentiated hiPSC-CMs were measured using RT-PCR. Briefly, cellular genomic RNA and cDNA were prepared using the Cell to cDNA kit (Invitrogen, Carlsbad, U.S.A.) according to the manufacturer’s instructions. Corresponding primers were designed, and RT-PCR was performed according to the manufacturer’s protocol.

### Cardiomyocyte hypertrophy model

hiPSC-CMs were uniformly seeded into 24-well plates and divided into either the control or hypertrophic cardiomyocyte (HC) group. For cardiomyocyte hypertrophy induction, hiPSC-CMs were incubated with 10 nM ET-1 for 24 h. A group of cells was also treated with 10 μm of the ET-1 inhibitor Bosentan (Selleck, U.S.A., Cat: S305102) 24 h following ET-1 incubation. The cell culture medium was changed every 24 h. Expression of brain natriuretic peptide (BNP) (Abcam, U.K., Cat: ab236101) and α-actinin (Cell Signaling, U.S.A., Cat: #3134S) was detected using immunofluorescence. The ratio of MYH7/MYH6T in the HC group was determined using real-time PCR. Briefly, total RNA was extracted, and the corresponding cDNAs were reverse transcribed according to the instruction provided by the GoScriptTM Reverse Transcription System kit (Promega; Madison, Wisconsin, U.S.A.). MYH7 and MYH6 expression were detected using real-time fluorescent quantitative PCR using the QuantStudio 3 Real-Time PCR System (Thermo Fisher Scientific, U.S.A.). The mean RT-qPCR threshold (*C*_t_) values of the reference gene (GAPDH) and target genes (MYH7 and MYH6) was calculated, and the ratio of MYH7/MYH6 was estimated. The primers were designed as follows: GAPDH-F: 5′-ACAGCAACAGGGTGGTGGAC-3′; GAPDH-R: 5′-TTTGAGGGTGCAGCGAACTT-3′; MYH6-F:5′-CCCGATGACAAGGAAGAGTTTG-3′; MYH6-R:5′-ATCTTGTCGAACTTGGGTGG-3′; MYH7-F:5′-AGTTCACACGCCTCAAAGAG-3′; MYH7-R: 5′-TCTGCCAGGTTGTCTTGTTC-3′.

### Safety evaluation of puerarin on cardiomyocytes

The culture medium was aspirated from the matrix coated 96-well platinum plates (E-Plate 96 form ACEA Biosciences, San Diego, CA, USA) and then we added 50 μl of myocardial medium. Cells were cultured at 5% CO_2_ at 37°C and then processed using real-time label-free cell analysis (RTCA) with the xCELLigence® RTCA MP instrument (ACEA Biosciences) to determine the baseline volume. Cardiomyocytes were seeded at 30,000 cells/well onto the pretreated 96-well platinum plates. The medium was changed every other day until the cells reached steady state (∼3–4 days). Different concentrations of puerarin were then added to the wells. RCTA was continuously performed for 48 h.

### Patch-clamp

#### Action potential measurement

A capillary glass tube (Sutter Instruments, Novato, CA, U.S.A.) was stretched to form a recording electrode using a microelectrode puller (Sutter Instruments). The MP285 Microelectrode Manipulator (Sutter Instruments) was operated under an AE31 TrinocularAE30 Inverted Microscope (Motic, BC, Canada) to expose the recording electrode to the cells, and vacuum suction was applied to form a GΩ seal. Once the GΩ seal was formed, rapid capacitance compensation was performed. The negative pressure was continued, and the cell membrane was suctioned for whole cell recording. We compensated for the slow capacitance and recorded the film capacitance and series resistance. No leakage compensation was applied, and changes in the action potential parameters were recorded.

The current recording scheme for action potential measurement was as follows: the current recorder [EPC-10 USB amplifier (HEKA)] was switched to the current clamp mode once the whole cell was sealed, and the cellular membrane current was clamped at 0 pA. The current was gradually decreased in 100 pA increments and recording continued until the action potential was excited. The test drug was then added, and the current was continuously recorded for 3 min. All data were stored in the PatchMaster (HEKA, Lambrecht, Germany) software. The extracellular solution used for patch clamping consisted of 140 mM NaCl, 10 mM HEPES, 3.5 mM KCl, 2 mM CaCl_2_, 1 mM MgCl_2_, 10 mM glucose, and 1.25 mM NaH_2_PO_4_, at pH 7.4. The pipette solution used for patch clamping consisted of 140 mM K-gluconate, 5 mM NaCl, 0.1 mM CaCl_2_, 1 mM MgCl_2_, 10 mM HEPES, 1 mM EGTA, and 2 mM Mg-ATP, at pH 7.2 (KOH adjusted).

#### Sodium ion channel measurement

IV-curve: The cell membrane potential was initially maintained at −90 mV, and then stepped from −120 to 100 mV in 5 mV step increments. Activation-curve: The cell membrane potential was initially maintained at −90 mV and then stepped to −120 mV for 200 ms. The cell membrane potential was then stepped from −80 to 100 mV in 5 mV step increments to detect the activation state of the channel. Inactivation-curve: The cell membrane potential was initially maintained at −90 mV, then stepped from −120 to 40 mV in 5mV step increments for 1000 ms, and then continuously stepped to 0 mV for 50 ms.

#### Sodium current detection

Recovery-curve: The cell membrane potential was initially maintained at −90 mV, then stepped to −10 mV for 250 ms to inactivate the sodium current, then stepped to −90 mV to restore the sodium current. Finally, the cell membrane potential was stepped to −10 mV for 250 ms to detect the sodium current.

During recordings of sodium current, the pipette solution contained 120 mM CsCl, 1 mM MgCl_2_, 10 mM HEPES, 4 mM Mg-ATP, 10 mM EGTA, 0.3 mM Na_2_-GTP, at pH 7.2 (adjusted with CsOH). The extracellular solution consisted of 140 mM NaCl, 3.5 mM KCl, 2 mM CaCl_2_, 1 mM MgCl_2_, 10 mM HEPES, 10 mM glucose, 1.25 mM NaH_2_PO_4_ 1, 0.1 mM CdCl, 1 mM 4AP, at pH 7.4 (adjusted with NaOH).

### Cell groups

The experiment was divided into five groups: control group, HC group (10 nM ET-1 treatment for 24 h), puerarin control group (200 μg/ml treatment for 12 h), puerarin pre-treatment group (PPr) (pre-treatment with 200 μg/ml puerarin for 12 h followed by 10 nM ET-1 induced cardiac hypertrophy), and puerarin post-treatment (PPo) group (10 nM ET-1 induced cardiac hypertrophy, followed by 200 μg/ml puerarin for 12 h). Eight cells were independently tested in each group during the recording period, and all electrophysiological experiments were performed at 32–35°C.

### Statistical analysis

Numerical data are presented as mean ± standard error of the mean (SEM). Comparisons were performed using an unpaired Student’s *t*-test (two-tails) and one-way ANOVA followed by Tukey’s post-test. Each data point was repeated from 8 to 10 independent samples. A *P* value < 0.05 was considered significant.

## Results

### Differentiation and purification of hiPSC-CMs

**Figure 1:**
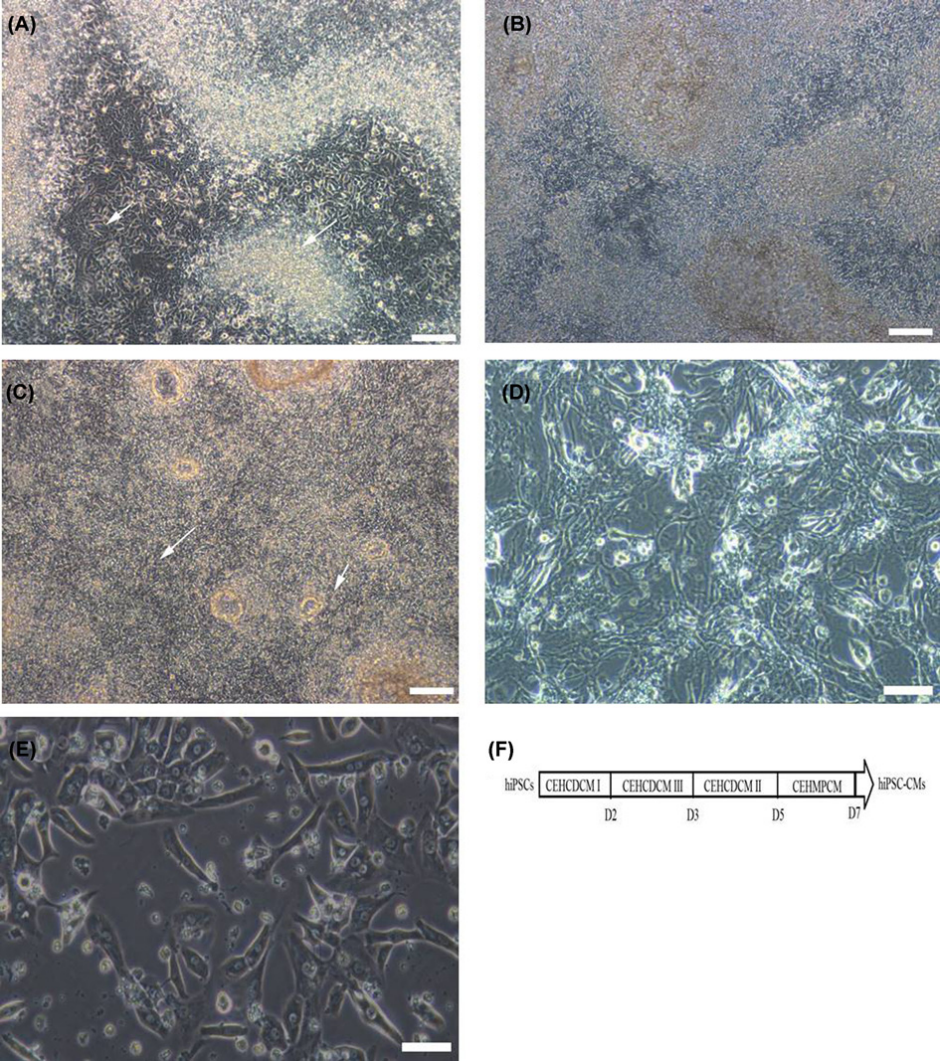
Differentiation and purification of hiPSC-CMs (**A**) After culturing with CEHCDCM I medium for 48 h, the hiPSCs showed typical deformation (short arrow) and a large number of mesodermal cells migrated out from the clones (long arrow); scale bar: 300 μm. (**B**) Following incubation with CEHCDCM III for 24 h, the mesoderm cells continued to grow and increase in density; scale bar: 300 μm. (**C**) After incubation with CEHCDCM II for another 48 h, a large number of mesodermal cells differentiated into cardiomyocytes (hiPSC-CMs, with typical myocardial morphology) (short arrow) and began to beat (long arrow); scale bar: 300 μm. (**D**) The differentiated hiPSC-CMs were purified in CEHMPCM, and the non-cardiomyocytes began to die; scale bar: 100 μm. (**E**) The differentiated hiPSC-CMs were stable after incubation with CEHMMM for 24 h; scale bar: 100 μm. (**F**) Figure shown a schedule of cardiomyocytes (hiPSC-CMs) differentiation from hiPSCs. CEHCDCM, CEHCDCM I, CEHCDCM II, and CEHCDCM III were different differentiation mediums purchased from CELLAPY (Beijing, China). D1, D3, D5, and D7 represented day 1,3, 5, and 7, respectively.

hiPSCs cultured with CEHCDCM I for 48 h displayed typical morphological deformation, and a large number of mesodermal cells migrated out from the clones (Figure 1A). After culturing the cells with CEHCDCM III for 24 h, the mesoderm cells continued to grow and increased in density (Figure 1B). After incubation with CEHCDCM II for 48 h, a large number of mesoderm cells differentiated into cardiomyocytes, displaying specific cardiomyocyte morphology and functional properties (Figure 1C). The differentiated cardiomyocytes were purified with CEH MPCM, while the non-cardiomyocytes gradually died (Figure 1D). The differentiated cardiomyocytes were maintained in CardioEasy Human Myocardium Maintenance Medium (CEHMMM) for 24 h. The final purity of the differentiated cardiomyocytes exceeded 95% (Figure 1E). A timeline for hiPSC-CMs culture is shown in Figure 1F.

**Figure 2:**
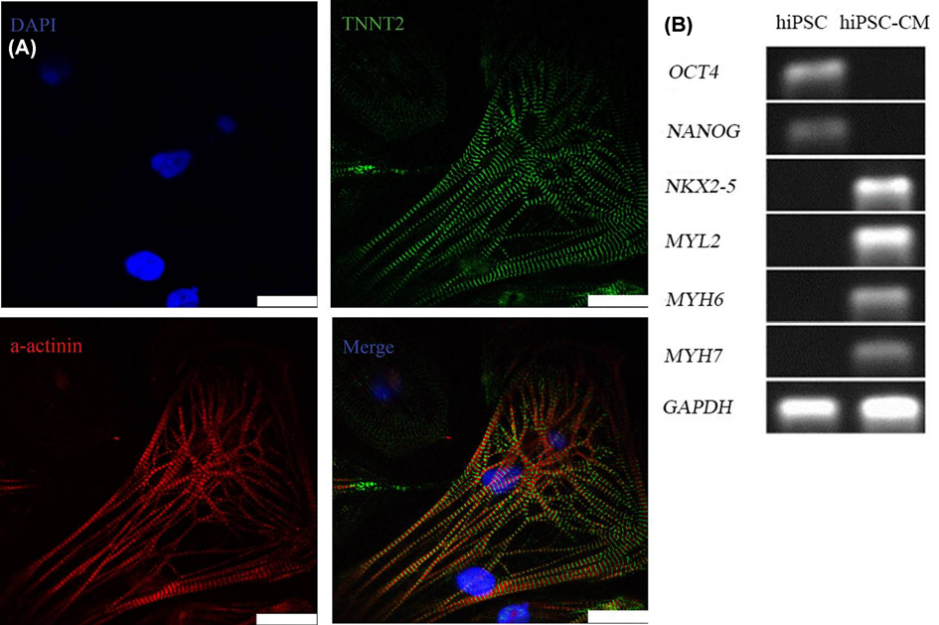
Identification of the differentiated hiPSC-CMs (**A**) Immunofluorescence staining of hiPSC-CMs with α-actinin (green, right-up) and TNNT2 (red, left-down) antibodies. Nuclei were stained using DAPI (blue, up-left); scale bar: 25 μm. (**B**) Gene expression of pluripotency-related genes (OCT4 and NANOG) and myocardial specific genes (NKX2-5, MYL2, MYH6, and MYH7) was measured using RT-PCR. GAPDH expression was used as the reference gene.

The differentiated cardiomyocytes were then identified using immunofluorescence detection of cardiomyocyte markers α-actinin and TNNT2. The cells stained positively for both α-actinin and TNNT2 (Figure 2A). Real-time PCR showed that the pluripotency-related genes NANOG and OCT4 were expressed in the hiPSCs; however, cardiomyocyte-related genes, including NKX2-5, MYL2, MYH6, and MYH7, were not detected. Compared with the hiPSCs, all of the cardiomyocytes-related genes (NKX2-5, MYL2, MYH6, and MYH7), but not the pluripotency genes (OCT4, NANOG) were expressed in the differentiated cardiomyocytes (hiPSCs-CMs) (Figure 2B).

### Preparation of HC model

**Figure 3:**
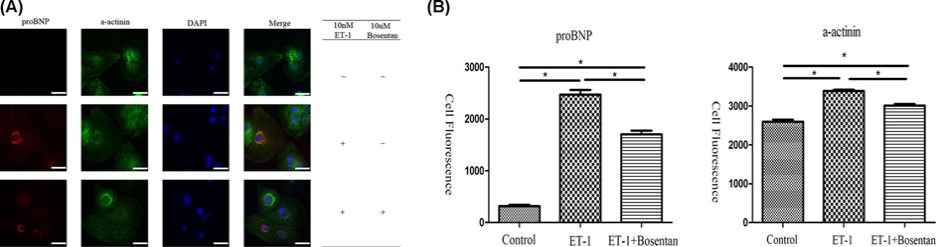
Immunofluorescence staining of the ET-1-induced cardiomyocytes (hiPSC-CM) (**A**) After induction of hiPSCs with 10 nM ET-1 (middle panels) for 24 h, expression of BNP (red) and α-actinin (green) were enhanced, and the cell shape was increased compared with the non-treated cells (up panels) and Bosentan (ET-1 antagonist) treated cells (lower panels); scale bar: 40 μm. (**B**) The levels of proBNP and α-actinin were significantly increased in the ET-1 group compared with the control group. However, Bosentan treatment reduced the expression of proBNP and α-actinin; however, both were still expressed at lower levels compared with the control group. *: *P* < 0.05 compared with control group.

**Figure 4:**
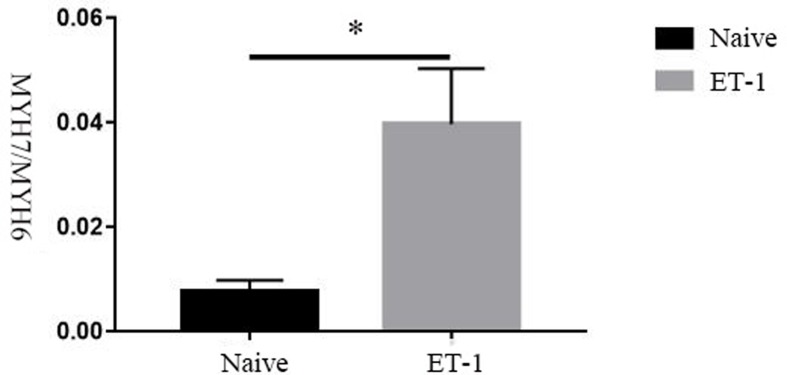
Alteration of MYH7/MYH6 ratio after ET-1 induction ET-1 induction significantly increased the ratio of MYH7/MYH6 in the hiPSC-CMs compared with the non-ET-1 treated cells (native) as measured by RT-PCR.

**Figure 5:**
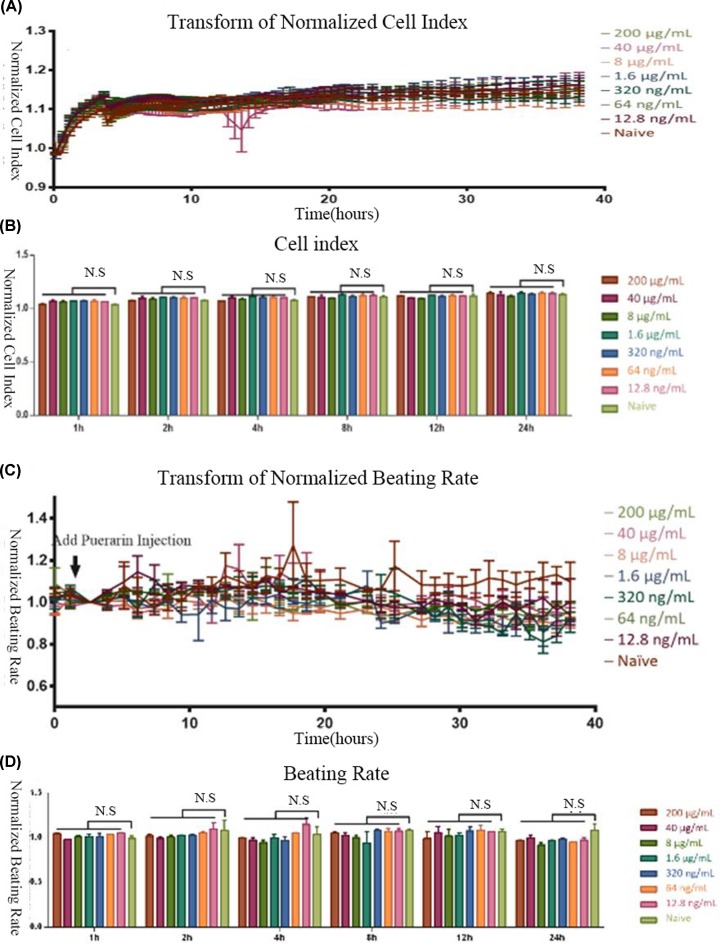
Toxic effect of serial concentrations of puerarin on cardiomyocytes (**A**) Cell survival (CI) was detected under the indicated treatment concentrations of puerarin from 0 to 40 h. (**B**) Statistical analysis of the CI data in the groups treated with different concentration of puerarin at 0, 1, 2, 4, 8, 12, and 24 h. (**C**) The beating rate (BR) was recorded under the indicated treatment concentrations of puerarin from 0 to 40 h. (**D**) Statistical analysis of the BR data in the groups treated with different concentration of puerarin at 0, 1, 2, 4, 8, 12, and 24 h. N.S: *P* >0.05.

After induction of the hiPSCs with 10 nM ET-1 for 24 h, the cell morphology was indicative of hypertrophic cardiomyocytes and BNP and α-actinin expression was increased (Figure 3). Quantitative analysis of the fluorescence images showed that the expression of proBNP and α-actinin in the ET-1 group was significantly higher compared with the control group (*P* < 0.05). After treatment with Bosentan, the expression of proBNP and α-actinin was reduced compared with the ET-1 group (*P* < 0.05); however, the expression of these proteins was still higher compared with the control group (*P* < 0.05) (Figure 4). In addition, the MYH7/MYH6 ratio was significantly increased in the ET-1 treated cells compared with the control hiPSCs (*P* = 0.0422) (Figure 5). We found that the ET-1-treated hiPSCs differentiated into HCs (hiPSC-CMs), indicating that the cardiac hypertrophy model was successfully established. hiPSC-CM differentiation was ET-1 dependent since pre-treatment with Bosentan (an ET-1 antagonist) prevented hiPSC-CM differentiation (Figure 3).

### Safety evaluation of puerarin in cardiomyocytes

We evaluated the influence of serial concentrations of puerarin on cell index (CI) and beating rate (BR) of the hiPSC-CM using RTCA. We found that puerarin did not cause significant cytotoxicity in the hiPSC-CMs at the ∼200 μg/ml. No significant difference in CI was observed between the different puerarin and negative control (0 μg/ml) groups at each time point. BR slowed after 12 h of continuous observation. However, there was no correlation between functional effects and puerarin concentration (Figure 5). The highest concentration (200 μg/ml) of puerarin was 10 times greater than that normally found in the blood after 4 h of clinical application. Since this concentration did not cause any cytotoxic effect on hiPSC-CMs, we selected this concertation for use in subsequent experiments.

### Puerarin effects on cardiomyocyte action potential

**Figure 6:**
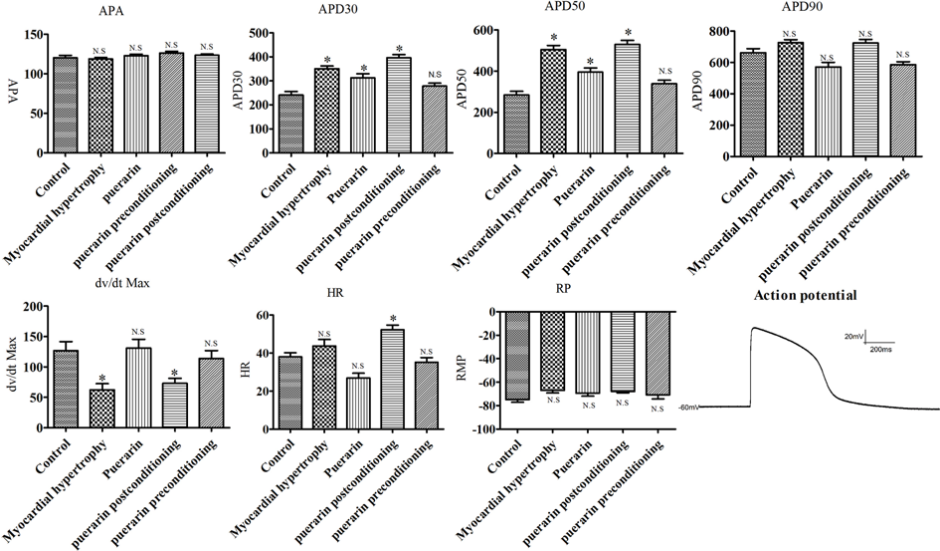
Activity of puerarin on action potential Action potentials were recorded in the indicated groups. APA: action potential phase 0 depolarization amplitude; APD30: depolarization start to repolarization 30% time; APD50: depolarization start to repolarization 50% time; APD90: depolarization start to repolarization 90% time; d*V*/d*t* Max (*V*_max_): maximum depolarization velocity in phase 0; HR: number of beats per minute in myocardium; RP: resting membrane potential; Action potential: action potential map of hiPSC-CMs. *: *P*<0.05 compared with the control group. N.S: *P* >0.05.

The HC and the PPo groups had slightly prolonged depolarization at 90% of the repolarization time (APD90), significantly elongated depolarization started at 50% of the repolarization time (APD50), and 30% of the repolarization time (APD30). In addition, treatment extended the overall time course of the cardiomyocyte action potential. There was no significant difference in the depolarization amplitude (APA) of the hiPSC-CMs action potential in phase 0 among the HC and PPo groups. The maximal depolarization velocity (d*V*/d*t* Max or *V*_max_) in phase 0 was significantly reduced. The resting potential (RP) of the hi-PSC-CMs was slightly reduced and the frequency of spontaneous pulsation of the cells was accelerated. The puerarin and the PPr groups had a slightly shortened APD90, but prolonged APD50 and APD30, which slightly curtailed during the course of the full action potential. APA was not significantly different among the groups and there was no effect on *V*_max_ and RP, but the spontaneous beat frequency of the cardiomyocytes slowed (Figure 6).

### Kinetic influence of puerarin on the sodium ion channel

**Figure 7:**
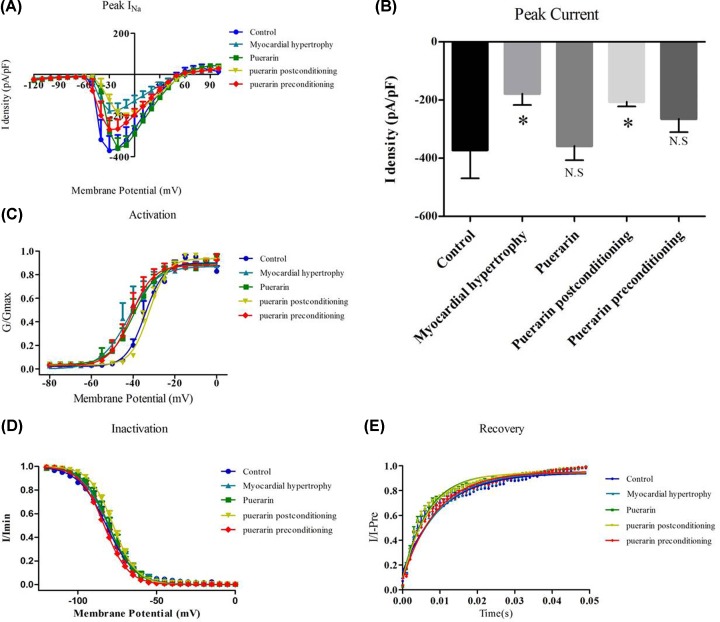
Electrophysiology measurements of the sodium ion channel (**A**) IV curves: cell membrane potential in the indicated groups was initially maintained at −90 mV, and then gradually stepped from −120 to −100 mV in 5 mV step intervals. (**B**) The average of the peak currents in each group. (**C**) Activation curves of sodium channel: cell membrane potential in the indicated groups was initially maintained at −90 mV, and gradually stepped to −120mV for 200 ms, and then stepped from −80 to 100 mV in 5 mV step intervals to detect the activation state of the channel. (**D**) Inactivation curves of sodium channel: cell membrane potential in the indicated groups was initially maintained at −90 mV, and then gradually stepped from −120 to 40 mV for 1000 ms, then stepped to 0 mV for 50 ms in 5 mV step intervals to detect sodium current. (**E**) Recovery curves of sodium channel: cell membrane potential in the indicated group was initially maintained at −90 mV, and then gradually stepped to −10 mV for 250 ms to inactivate sodium current, then stepped to −90 mV for different durations to recover the sodium current, and then finally stepped to −10 mV for 250 ms to detect the sodium current. Groups: control (●), myocardial hypertrophy (▲), puerarin (▪), puerarin postconditioning (▼), and puerarin preconditioning (♦). *: *P* < 0.05 or NS: *P* > 0.05 compared with the control group.

**Figure 8:**
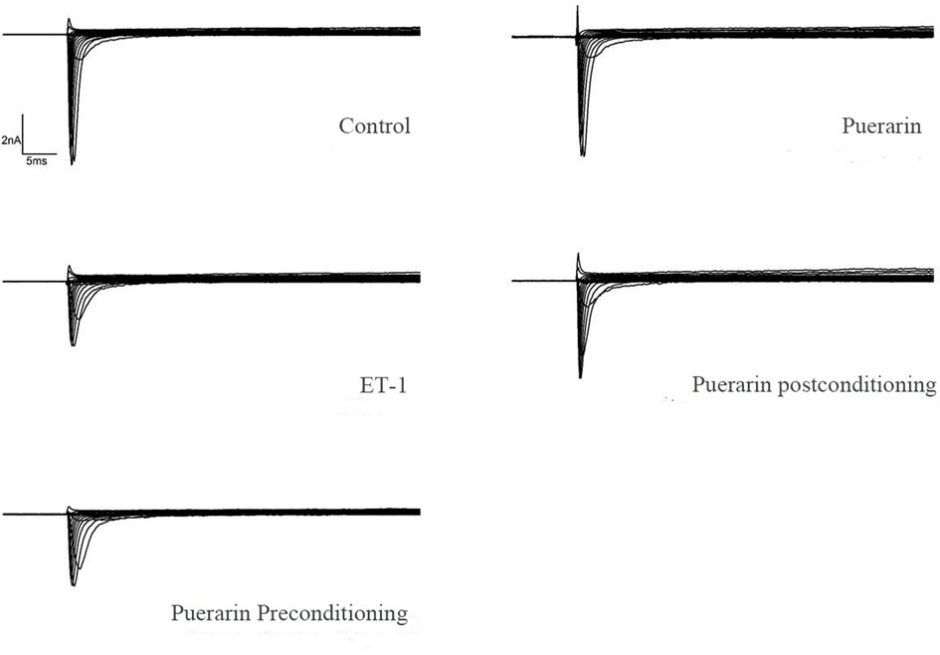
Typical current trace of sodium channel Cellular membrane potential was maintained at −90 mV, and then increased at 5 mV interval in the control, puerarin, ET-1, puerarin preconditioning, and puerarin popstconditionaing group, respectively. Once the cellular membrane potentials stepped to 100 mV, the current mode of *I*_Na_ was recorded.

The I-V curve and sodium ion channel peak current density column were recorded in the control, HC, puerarin, PPr, and PPo groups. The original records of the *I*_Na_ are shown in Figure 7. A typical current trace of the sodium channel is shown in Figure 8. Compared with the control group (−371.7 ± 97.76), the peak current density of the sodium channel in the HC group (−178.5 ± 38.57) was significantly decreased, indicating that the sodium channel was inhibited by ET-1. There were no significant sodium channel effects in the puerarin group (−357.9 ± 48.76). Meanwhile, *I*_Na_ did not significantly improve in the PPo group (−205.6 ± 16.56) compared with the HC group. However, PPr (−265.0 ± 45.14) did improve the *I*_Na_ compared with the HC group. The recorded *I*−*V* curves and the current density histograms are presented in Figure 7A,B, respectively. With regards to the sodium channel activation curve, the half-activation voltage (V50) and the slope of the curve (Slope) were not significantly different among the groups (Figure 7C). There were no significant differences in the V50 and slope in the sodium channel inactivation – curve among the groups (Figure 7D). There were no significant differences in the sodium channel recovery time (Tau) among the groups (Figure 7E). Comparison of the activation curve, inactivation curve V50, Slope, and recovery curve (Tau) between each group are shown in Table 1.

**Table 1:** Average data of activation, inactivation, and recovery curves of each group

		Control	Hypertrophy cardiomyocyte	Puerarin	Puerarin post-treatment	Puerarin pre-treatment
Act. Curv	V50	−34.40 ± 0.69	−42.22 ± 1.61	−39.68 ± 1.01	−32.34 ± 0.55	−41.00 ± 1.09
	Slope	4.54 ± 0.61	6.74 ± 1.47	5.91 ± 0.91	4.36 ± 0.48	5.26 ± 0.96
Inact.Curv	V50	−81.90 ± 0.60	−80.66 ± 0.52	−80.22 ± 0.43	−77.51 ± 0.24	−83.29 ± 0.37
	Slope	9.09 ± 0.53	8.56 ± 0.46	8.10 ± 0.38	7.45 ± 0.21	7.66 ± 0.33
Rec. Curv	Ta (ms)	8.869−11.39	9.023–10.49	6.326−7.156	6.529−7.336	8.509−9.608

Act. Curv: Activation curve; Inact. Curv: Inactivation curve; Rec. Curv: Recovery curve; V50 and Slope are expressed as mean ± standard deviation; Tau is expressed in 95% confidence interval. The activation curve, V50, Slope of the inactivation curve, and the Tau of the recovery curve of each group were not statistically different compared with the control group.

## Discussion

Here, we demonstrated the ability to differentiate hiPSCs into cardiomyocytes (hiPSC-CMs) using a series of CEHCDCM cell culture mediums. The typical characteristics of the hiPSC-CMs were confirmed by: (1) immunofluorescence staining of cardiomyocyte markers, α-actinin, and TNNT2; (2) comparing expression of pluripotency-related genes (OCT4 and NANOG) and negative expression of cardiomyocyte-related genes (NKX2-5, MYL2, MYH6 and MYH7); (3) measuring spontaneous cardiomyocyte beats 10 days following differentiation onset; and (4) recording action potentials in the spontaneous beating cells.

Inherently, there are significant differences between experimental animal model cardiomyocytes and human cardiomyocytes. For instance, the number of beats per minute in the human myocardium are significantly different compared with rat, mouse, and rabbit myocardium. Our hiPSC-CM model displayed standard electrophysiological and biochemical properties of normal human cardiomyocytes, which can be stimulated by electrical excitation. Therefore, hiPSC-CMs are a good cellular model to investigate a variety of biochemical and electrophysiological properties in cardiomyocytes [14].

The cardiomyocyte hypertrophy model can be induced by angiotensin II, isoproterenol, growth hormone, insulin-like growth factor-1, and ET-1. Among them, isoproterenol is a synthetic chemical that is not naturally produced in the human body, while angiotensin II is a circulating hormone that does not increase significantly in the local myocardium. Growth hormone and insulin-like growth factor-1 can induce cardiac hypertrophy *in vitro* and in animal experiments [20], yet its concentration in humans is not high enough to cause cardiac hypertrophy. ET-1 can be synthesized by stimulated vascular endothelial cells and cardiomyocytes. ET-1 can accumulate in myocardial tissue through autocrine or paracrine mechanisms, leading to a series of pathophysiological processes, such as cardiac hypertrophy [5]. Therefore, the ET-1-indcued HC model that we used in the present study most closely mimicked the pathological state of human cardiac hypertrophy and is therefore an ideal model for drug testing.

Puerarin has been shown to influence ion channels in animal cardiomyocytes by inhibiting the inward rectifier potassium channel [8], activating the calcium-sensitive potassium channel [9], and blocking the calcium channel [10]. Most studies investigating the activity of puerarin on myocardial ion channels have generally been performed in animal-derived cardiomyocytes. Thus, the effects of puerarin on ion channels in human cardiomyocytes have not been reported. Experiments directly performed in human cardiomyocytes could closely represent human pathophysiology and therefore would be more valuable for developing future clinical applications to treat arrhythmias.

The patch clamp technique entails creating a tight seal between a microelectrode and a cellular membrane that allows one to record the electrical activity of ion channels with voltage or current clamping. In 1980, Dr Sigwrth and Dr Neher used a vacuum suction on the recording electrode to generate a high-resistance seal of 10–100 GΩ between the cell membrane and microelectrode. This method greatly improved the patch clamp technique, which provided a reliable and sensitive technology to investigate ion channel function [21]. Therefore, we used the whole-cell patch clamp method to study the action potential and sodium current of hypertrophic cardiomyocytes. The recorded action potentials of the hiPSC-CMs in the present study were divided into five periods with patterns identical with those of other reported human ventricular myocardium action potentials [22]. The results showed that the APD50 and APD30 of HCs and the overall action potential duration were prolonged compared with normal cardiomyocytes. The APA in phase 0 did not change; however, the *V*_max_ was significantly reduced. PPo did not eliminate or reduce the above effects. Although the PPr group had no effect on APD50 and APD30 in HCs, it did improve *V*_max_ in phase 0. Since the depolarization of ventricular myocytes in phase 0 is closely related to the influx of sodium ions, we next focused on the parameters of sodium ion channels.

The functional states of ion channels can be classified into resting state, activated state, and inactive state. In the resting state, the ion channel is closed. The ion channel will enter the active state from the resting state when the membrane potential changes, and at this moment the ion can pass through the cell membrane. This ion influx will change the membrane potential and cause the ion channel to enter the inactive state, which in turn will close the ion influx through the cell membrane. Along with constant depolarization, the ion channel re-enters the resting state. To determine the effect of puerarin treatment on sodium channels in HCs, we assessed the *I–V* curve, activation curve, inactivation curve, and recovery curve after inactivation of the sodium channel. Among these, the *I–V* curve reflects the influence of external factors on ion selectivity and rectification characteristics of the channel. The activation curve, the inactivation curve, and the post-inactivation recovery curve comproise the ion channel voltage-dependent gating characteristics. During myocardial depolarization, sodium ions flow through the sodium ion channel into the cell along the chemical gradient, which is the main inward current that plays a crucial role in the rapid rise of the membrane potential and the action potential. The gene encoding the sodium channel of human cardiomyocytes arises from the third pair of chromosomes, called SCN5A, and its abnormal expression can lead to congenital long QT syndrome, Brugada syndrome, and tachyarrhythmia [23]. Our experimental data indicated that the peak density of *I*_Na_ decreased after myocardial hypertrophy, but the shape of the *I–V* curve remained unchanged, suggesting that the ion channel selectivity and rectification characteristics did not change. *I*_Na_ is closely related to phase 0 of the action potential, and the decrease in current density is an important underlying cause of the decrease in *V*_max_. This change can lead to impaired impulse conduction and the formation of excitatory re-entry arrhythmias [24]. We found that PPo did not increase the peak density of *I*_Na_; however, PPr elevated the peak density of *I*_Na_. Therefore, PPr can increase the rate of action potentials in the 0 phase and accelerate sodium ion inflow and impulse conduction to prevent excitatory re-entry arrhythmias. In addition, PPr stabilized the electrophysiological properties of cardiomyocytes and reduced the heterogeneity between cardiomyocytes. The preventive activity of puerarin on myocardial depolarization and repolarization dispersion reduced the incidence of re-entry and delayed depolarization, as well as decreased the occurrence and progression of ventricular hypertrophy arrhythmia. Interestingly, there was no significant effect on the activation, inactivation, and recovery of the sodium channel among the groups, indicating that the voltage-dependent gating characteristics of the sodium ion channel did not significantly change.

## Conclusion

A human hypertrophic cardio myocyte model was successfully obtained. Patch-clamp study indicated that pre-treatment with puerarin can improve the depolarization rate of the hypertrophic cardio myocytes by increasing the peak density of *I*_Na_ current. This result suggested that puerarin has a certain preventive effect on re-entrant excitation arrhythmia as well as positive significance on the electrophysiological stability of hypertrophic cardio myocytes.

## Data Availability

The datasets generated and analyzed during the present study are available from the corresponding author upon reasonable request.

## Abbreviations

APA: depolarization amplitude
BNP: brain natriuretic peptide
BR: beating rate
CEHCDCM: CardioEasy human cardiomyocyte differentiation complete medium
CEHMMM: CardioEasy human myocardium maintenance medium
CEHMPCM: CardioEasy human myocardial purified complete medium
CI: cell index
d*V*/d*t* Max or *V*_max_: maximal depolarization velocity
ET-1: endothelin-1
HC: hypertrophic cardiomyocyte
hiPSC: human induced pluripotent stem cell
hiPSC-CM: hiPSC-cardiomyocyte
JNK: Jun N-terminal kinase
KLM4: Kruppel-like factor 4
MAPK: mitogen-activated protein kinase
MYH6: α-cardiac myosin heavy chain
MYL2: myosin light chain 2
*I*_Na_: sodium current
NKX2-5: NK2 homeobox 5
OCT4: octamer-binding transcription factor 4
PKC: protein kinase C
PLA2: phospholipases A2
PLC: phospholipase C
PPo: puerarin post-treatment
PPr: puerarin pre-treatment
RP: resting potential
RTCA: real-time label-free cell analyzer
SOX2: sex determining region Y-box 2
TNNT2: troponin T2
*V*_max_: maximum depolarization velocity
